# The Evolution of Molecular Compatibility between Bacteriophage ΦX174 and its Host

**DOI:** 10.1038/s41598-018-25914-7

**Published:** 2018-05-29

**Authors:** Alexander Kula, Joseph Saelens, Jennifer Cox, Alyxandria M. Schubert, Michael Travisano, Catherine Putonti

**Affiliations:** 1 Department of Biology, Loyola University Chicago, Chicago, IL USA; 2 Bioinformatics Program, Loyola University Chicago, Chicago, IL USA; 3 Department of Ecology, Evolution and Behavior, University of Minnesota, Saint Paul, MN USA; 4 BioTechnology Institute, University of Minnesota, Saint Paul, MN USA; 5 Department of Computer Science, Loyola University Chicago, Chicago, IL USA; 6 Present Address: Department of Biology, University of Miami, Coral Gables, FL USA; 7 Present Address: Department of Molecular Genetics and Microbiology, Duke University, Durham, NC USA; 8 Present Address: Division of Bacterial, Parasitic, and Allergenic Products, Office of Vaccines Research and Review, Center for Biologics Evaluation and Research, U.S. Food and Drug Administration, Silver Spring, MD USA

## Abstract

Viruses rely upon their hosts for biosynthesis of viral RNA, DNA and protein. This dependency frequently engenders strong selection for virus genome compatibility with potential hosts, appropriate gene regulation and expression necessary for a successful infection. While bioinformatic studies have shown strong correlations between codon usage in viral and host genomes, the selective factors by which this compatibility evolves remain a matter of conjecture. Engineered to include codons with a lesser usage and/or tRNA abundance within the host, three different attenuated strains of the bacterial virus ФX174 were created and propagated via serial transfers. Molecular sequence data indicate that biosynthetic compatibility was recovered rapidly. Extensive computational simulations were performed to assess the role of mutational biases as well as selection for translational efficiency in the engineered phage. Using bacteriophage as a model system, we can begin to unravel the evolutionary processes shaping codon compatibility between viruses and their host.

## Introduction

The spread of emergent viral diseases critically depends upon rapid adaptation to novel hosts^1^. Analogous to that observed in natural populations (e.g., ref.^2^), experimental evolution of viral pathogens has also demonstrated rapid adaptation to new hosts^3^. Nevertheless, the molecular basis of host specificity within viruses remains contentious^4–8^. While selection experiments have shown that single nucleotide changes can be sufficient to facilitate a viral host shift (e.g., ref.^9^), bioinformatic surveys repeatedly show a high degree of genomic correspondence between viral pathogens and their hosts^4,10^. This is most evident within bacteriophage (phage) species^7,11^. For instance, codon usage of coliphages generally reflects the biased usage of their host which itself reflects the most abundant cognate tRNAs available within host cells^12–14^ and mRNA levels^15^. This correspondence of phage and host codon usage is not surprising given that viruses are frequently, often entirely, reliant on their hosts for biosynthesis. This dependency engenders strong selection for virus genome compatibility with potential hosts, a necessity for a successful infection.

While once referred to as “silent”, we now know that synonymous mutations can have a profound effect on both an organism’s phenotype and fitness^16–18^. Deviations from neutral expectations of codon usage can be the result of selection for translational efficiency and/or accuracy, mutational biases, drift, control of gene expression, and structure^19–28^. Reduced viral fitness has been detected in molecular engineering of viral codons via synonymous mutations^29–41^ (also see reviews^42,43^). These fitness losses are largely attributed to a reduction of genome translation and show that codon engineering is a promising avenue for generating new vaccines^29–33,36–43^. While the immediate cost of synonymous mutations on viral fitness has been observed, causes and consequences of sequence-specific host adaptation remains elusive.

Phages provide an ideal model system for exploring the evolution of codon usage bias. The literature is rich with experimental evolution of phages, applying various forms of selection^44–50^. Furthermore, substantial bioinformatic analysis of codon usage within phage^7,11^ and bacterial^19^ genomes has been conducted. Through experimental evolution of the codon-based attenuated T7 phage, fitness recovery was observed by evolutionary changes in codon use^34^. More rapid rescue has been observed in the passage of codon deoptimized eukaryote-infecting viruses having smaller RNA-based genomes^35,36^. The effects of codon deoptimization on fitness and the recovery of fitness, however, varies from one virus to the next^51^. As prior evidence has shown, synonymous mutations specifically introduced in species having small, compact genomes can have a profound impact on a species’ fitness^52–54^. The mechanisms that lead to pathogen-host genome compatibility remain uncertain, leaving the causal factors open questions.

We performed long-term experimental evolution to determine how and at what rate virus-host codon usage evolves, using engineered phage genomes. The coding sequence of the bacteriophage ΦX174 was targeted, replacing wild-type codons with deoptimized (relative to its host, *Escherichia coli* C) codons. Three different engineered strains, targeting two different coding regions within the ΦX174 genome, were created. The ΦX174 genome is small and compact, encoding for just 11 genes in the 5386 nucleotide ssDNA, circular genome; furthermore, ΦX174 is known to be sensitive to mutations^54,55^. The combination of engineered sequence changes allows for the simultaneous examination of the role of selection to affect sequence specific adaptation, specifically rates of reversion and translational efficiency within the *E. coli* host. Complementing our experimental efforts, extensive computational simulations were performed to assess the role of selection for translational efficiency. This multidisciplinary approach provides insight into how genome compatibility arises.

## Results

### Conservation of host genomic compatibility within microviruses

Codon usage within homologous gene sequences of ΦX174 and its two known closest relatives (G4 and α3) was examined. Despite only modest sequence similarity, orthologs are similar in their usage of codons favored within the highly expressed genes (HEGs) of their host, *E. coli* (Supplementary Fig. S1). This is true not only of the RefSeq sequences, but also microviruses isolated from environmental samples (results not shown). Furthermore, this trend was also observed within the more distant relative of ΦX174: ΦMH2K. ΦMH2K, also a microvirus, infects *Bdellovibrio bacteriovorus*. The variance of the estimated translation rate between genes was statistically significant (p-value = 0.00009) while the variance between the species was not. Thus, the observed level of gene-host codon compatibility in these microviruses is conserved regardless of the host species. The homologous coding regions for the F and J coding regions include a codon usage most congruent to their respective host’s codon usage biases (Supplementary Fig. S1) and thus were selected for subsequent experimental examination.

### Strain engineering

A 66 bp region within the ΦX174 capsid protein F coding region and a 69 bp region within the core protein J coding region were re-engineered to include alternate codons, often codons less preferred by the host, such that both regions were comparably deoptimized relative to the ancestral strain (see Methods; summarized in Table 1). Engineered mutants were created from a ΦX174 strain in our lab which was well-adapted to the growth conditions employed in the selection experiments carried out here. Two engineered mutants, S and E, were created for the F protein coding region. The S strain contains eleven synonymous substitutions within the 22 codon region (Supplementary Table S1); nine were achieved by single third position changes, while the remaining two codon substitutions included two base changes (first and second position Leucine). The E strain contained these same eleven synonymous substitutions in addition to one nonsynonymous codon replacement (Supplementary Table S1); this particular codon was chosen as sequenced ΦX174 strains vary in the amino acid encoded (histidine or arginine). Similarly, a deoptimized sequence was designed for a 23 codon region in J, henceforth referred to as the J strain. The J strain contains twelve synonymous substitutions; all substitutions are achieved by single third position changes (Supplementary Table S2).

**Table 1:** Summary of strains created through codon engineering.

Strain Name	Gene Targeted	Engineering	% Deoptimized
S	F	11 synonymous mutations	29.3
E	F	11 synonymous mutations; 1 nonsynonymous mutation	32.9
J	J	12 synonymous mutations	29.0

The S and E strains were propagated for 35 transfers. While a single propagation of the S strain was performed, the E strain was propagated in quadruplicate. The J strains were propagated for 50 transfers, in triplicate. Four replicates of the Anc strain, the unaltered ancestral strain, were also propagated serving as a control. (Further details regarding the experimental design are included within the Methods.) To distinguish between the engineered genomic sequences and the evolved genomic sequences, the following notation will be used. The engineered mutant strains prior to propagation are referred to as the “S strain”, “E strain”, and “J strain” created from the ancestral “Anc strain”. The serially passaged S, E and J strains are denoted as the S, E, and J lines, collectively referred to as the engineered lines, with replicates denoted by number. The propagated Anc strain is henceforth referred to as the C1, C2, C3, and C4 lines. As anticipated, initial plating of the engineered strains created here showed a significant reduction in the number of successful infections relative to the propagated Anc strain, as measured by plaque forming units (PFU) and burst size (Supplementary Fig. S2).

### Responses to selection

**Figure 1:**
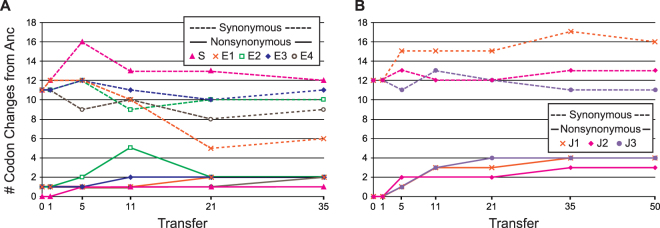
Number of synonymous and nonsynonymous codon differences between the engineered lines and the Anc strain over the course of the selection experiment: **(A)** F coding region engineered lines S and E1, E2, E3, and E4. **(B)** J coding region engineered lines J1, J2, and J3.

The targeted region was sequenced for all engineered lines from isolates collected after the 1^st^, 5^th^, 11^th^, 21^st^, and 35^th^ transfer; additionally, the J lines were sequenced after the 50^th^ transfer. Synonymous, as well as nonsynonymous, mutations occurred both for the codons that were initially manipulated as well as other codons in the region targeted in the engineered lines (Fig. 1). While the E2 line collected after the 11^th^ transfer shows the most nonsynonymous differences, five, by the next sampling many of these differences were no longer present in the population. Although several of the codons fixed within the engineered lines were those that were present within the Anc strain, this was not a general result. For each of the mutations identified, changes in codons were identified and assessed relative to the codon usage within the HEGs of *E. coli* C. For all engineered lines, the majority of the mutations result in a substitution for a codon more frequently used within *E. coli* C’s set of HEGs (shown in Supplementary Tables S1 and S2).

**Figure 2:**
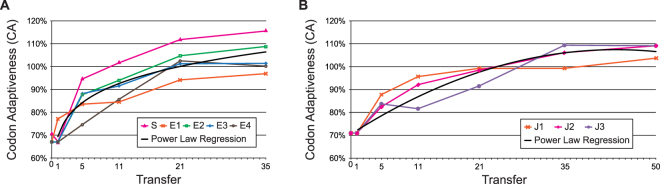
Codon adaptiveness (CA) of each of the engineered lines: (**A**) F coding region engineered lines S and E1, E2, E3, and E4. (**B**) J coding region engineered lines J1, J2, and J3.

**Figure 3:**
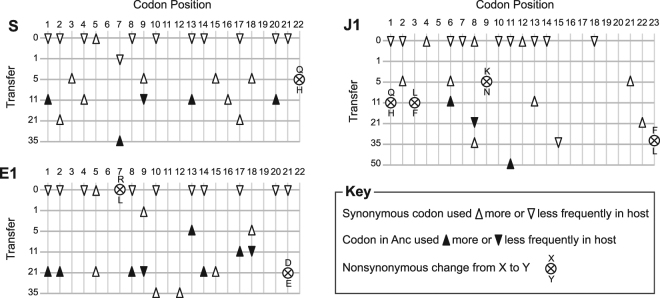
Codon changes observed within the engineered lines (relative to the codon in the Anc strain) over the course of the selection experiment. Synonymous mutations are indicated by triangles; those resulting in a codon more frequently used in the *E. coli* C HEGs are indicated by a “Δ” and the converse by a “∇”. If the mutation results in the codon present within the Anc strain, the triangle is solid black. Nonsynonymous mutations are indicated by ⊗.

To assess the putative effects of these mutations on the protein’s translational efficiency, we examined the codon adaptiveness (CA) of the targeted windows for each engineered line. This metric represents the individual engineered line’s usage of host-preferred codons relative to this same window in the Anc strain. Both the region within the F engineered lines as well as the region within the J engineered lines showed a consistent increase in CA over the course of selection (Fig. 2). The exception being the acquisition of a single synonymous mutation in the S line after 1 transfer and the J3 line after 11 transfers; the CA value of these lines, however, was rapidly improved by the next sampling. At the end of the selection experiment, seven of the eight engineered lines include codons which are utilized more frequently within the host’s set of HEGs than are present within this same window in the Anc strain (CA > 100%). Only the F coding region engineered line E1 did not exceed 100%. As the extension of the J lines for an additional 15 transfers reveals, the rate of change in the CA value diminishes (Fig. 2B). In parallel to the steady rise in the CA value, all of the engineered lines showed fitness improvements, with respect to both plaque formation and burst size (Supplementary Fig. S2). Figure 3 illustrates the individual mutations for three of the evolved lines; the remaining lines are shown in Supplementary Fig. S3 and a full listing of the mutations can be found in Supplementary Tables S1 (for the S and E lines) and S2 (for the J lines).

The simultaneous propagation of the C lines provides insight into the probability of mutations arising within the targeted regions of the engineered lines as a result of the selection experiment. The F and J protein coding regions were also sequenced for the C line after the 1^st^, 5^th^, 11^th^, 21^st^, 35^th^, and 50^th^ transfers. No nonsynonymous mutations were detected within the J protein coding region. One was observed within the F protein coding region, at genome position 1727 (L242F). This nonsynonymous mutation was first detected after the 21^st^ transfer and became fixed in the population; a synonymous mutation at this same position was detected as early as the 5^th^ transfer. This nonsynonymous mutation, however, is not unique to the evolved line; of 67 publicly available genomes in GenBank (Supplementary Table S3), 15 have Leucine (including the Anc strain) while the remaining 52 have Phenylalanine at this position.

### Unraveling the selective forces increasing the lines’ CA

**Figure 4:**
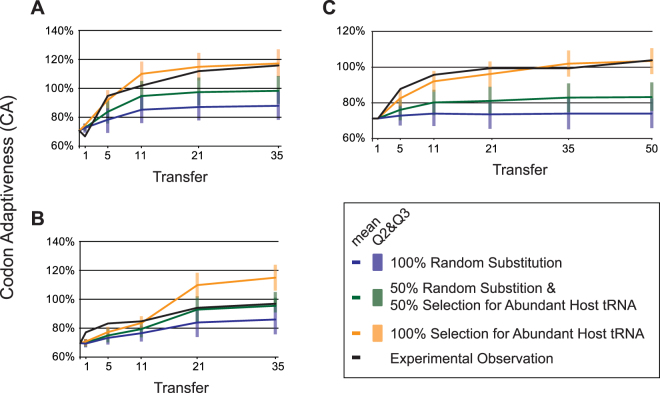
Average CA values predicted over time from simulations under three different variations of the role of translational selection and random mutation. The CA values from the experimental assays are shown by the black line. Each of the engineered lines is shown separately, (**A**) S, (**B**) E1, and (**C**) J1.

We investigated mutational effects using simulation. The simulation included the effects of random mutation and selection for translational efficiency (see Methods). Conducting 1000 replicates captured the landscape of mutations which could be explored by each engineered sequence. Comparison of the simulations and the experimental assays are shown for the S, E1, and J1 engineered lines in Fig. 4. (The remaining lines can be found in Supplementary Fig. S4.) As the number of mutations increases, increasing divergence is observed in the average CA values for a sequence under strong selection for translational efficiency (the 100% Selection for More Abundant Host tRNA model in yellow) and in its absence (the 100% Random Substitution model in blue). Even when mutations are introduced randomly, the CA value increases because the engineered sequences were severely deoptimized; however, in no case was the random model sufficient to recover the observed increases in codon adaptiveness.

The simulations for the S and E lines suggest that selection for translational efficiency is important in shaping the codon usage of all five of the engineered lines. While the role of selection for translational efficiency between the selected lines may vary, it is not sufficient to explain the number of mutations, reversals, or dN/dS rate. The mutational dynamics in the E1 (Fig. 4B) and E2 and E3 (Supplementary Fig. S4) lines indicate that translational selection is unlikely to be the only factor shaping their codon usage, following the mixed model (shown in Fig. 4 in green). In contrast, the experimental results for the three engineered J lines (Fig. 4C and Supplementary Fig. S4) mirror the expectations under strong selection to utilize codons more frequently within the host’s set of HEGs (yellow lines). Thus, biosynthetic compatibility appears to be a significant source of selection for these lines. The S (Fig. 4A) and E4 (Supplementary Fig. S4) lines also suggest that translational selection plays an important role in shaping its codon usage, albeit not as strong as within the engineered J lines.

### Epistasis

**Figure 5:**
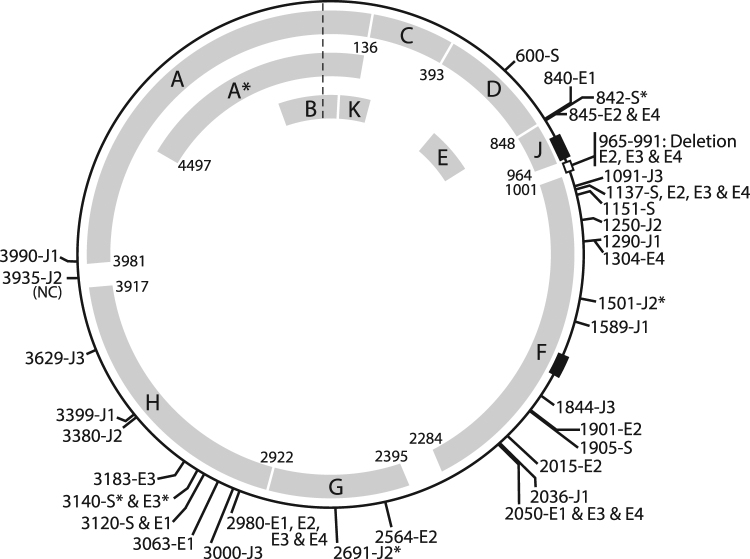
Compensatory mutations observed in all engineered lines. Asterisks indicate a synonymous mutation was observed; all other changes in a residue resulted in a nonsynonymous mutation. The open block indicates the location of the excision which occurred in the E2, E3 and E4 lines. The black blocks indicate the location of the 66 bp and 69 bp regions targeted within the S and E and J engineered strains, respectively. “NC” indicates a mutation within the noncoding region of the genome.

We hypothesized that there would be sequence changes in other coding regions over the course of selection as a result of protein-protein interactions. Complete genome sequencing was performed for the final isolates of all engineered lines as well as intermediate populations for the S and E lines (see Methods). Figure 5 illustrates the mutations identified within the S, E, and J engineered lines over the course of the selection experiment. The majority of the mutations observed occurred within the structural proteins, regardless of the region engineered. Three of the E lines (E2, E3, and E4) collected after the 35^th^ transfer include the excision of 27 nucleotides within the noncoding region between the J and F genes. In order to pinpoint when this excision occurred, isolates from the 22^nd^ through 34^th^ transfers were assayed for this excision via PCR of this region of the genome. The excision arose in the E2 line in the 27^th^ transfer, in the E3 line in the 29^th^ transfer, and in the E4 line in the 30^th^ transfer. A full listing of the mutations observed outside of the engineered regions throughout the course of the selection experiment within the eight engineered lines can be found in Supplementary Table S4.

By comparing the genetic variability of extant ΦX174 populations (Supplementary Table S3) with the experimental populations evolved here revealed shared diversity. Five of the nonsynonymous mutations within the engineered lines (four within the S and E lines and one within the J lines) (Supplementary Table S4) and all of the nonsynonymous mutations within the control lines (C1, C2, C3, and C4) (Supplementary Table S5) have previously been detected. In addition, through sequence analysis we find that many of these sites are highly variable, in particular the mutations observed within the control lines (Supplementary Table S5), such that numerous different codons and amino acids are exploited in viable strains. Furthermore, the 27 nucleotide excision observed within the E2, E3, and E4 lines is not unique; complete genomes in GenBank also contain this deletion. In fact, there is a correspondence between one of these mutations, A16V in the H protein coding region, and the deletion, both in our lines and those available from NCBI. The remaining nonsynonymous mutations within the engineered lines have not been previously detected within the genomes examined.

## Discussion

Through direct molecular manipulation, we investigated codon bias as it evolved. The bacteriophage ΦX174 was genetically engineered to have non-optimal codons resulting in low fitness. Over the course of the selection experiment, however, fitness increased in parallel with the incorporation of more host preferred codons (Fig. 1, Supplementary Fig. S2). Both synonymous and nonsynonymous mutations were observed within the targeted regions as the engineering of synonymous substitutions likely permitted the evolving virus to explore alternative paths within sequence space. This theory and the emergence of novel nonsynonymous mutations within the evolved engineered lines is not exclusive to our study; similarly, throughout the passage of codon deoptimized HIV strains novel nonsynonymous mutations were often frequent^35^. By targeting two individual coding regions of ΦX174 – the F and the J coding regions – we were able to determine that the response in phage-host codon compatibility observed was not a result of a particular gene or region selected. The engineered lines fixed between two (line J1) to six (lines E1, E3, and E4) reversions to the un-engineered ancestral codon. However, the reversions were almost exclusively divergent across lineages, with an exception of a single reversion, codon position 1, within the lines in which the F coding region was targeted (Fig. 3). Across the evolving lines, the majority of mutations within the targeted regions were for codons more frequently used within the host’s HEGs (Fig. 3). These results demonstrate parallel evolution in a molecular trait: phage-host codon compatibility.

Comparing codon usage within the individual targeted windows between the engineered lines and the Anc strain, we observed an increase in codon adaptiveness over the course of the selection experiment (Fig. 2). Much of the recovery of codon adaptiveness occurred early in the selection experiment regardless of the region targeted. Virtually all improvement in codon adaptiveness occurred within 21 transfers across all evolving lineages, and five of the lines (S, E1, E2, E3, and J1) had ~50% improvements within the first 5 transfers. The increase in CA was not restricted to evolution of the engineered codons, but also involved other codons in the targeted region, indicating that selection was not specific to those codons that were initially engineered. The evolved lines presented here provide empirical evidence that attenuation via codon deoptimization is not permanent, congruent with prior assessments of similar studies^51^.

Caution is however necessary in interpreting the evolutionary basis for increases in codon adaptiveness. The engineered sequence was severely codon deoptimized, and many mutations could have resulted in an increased CA value. The simulations performed under a strictly random substitution model (Fig. 4, blue lines) capture this consequence; the average final CA recovery under this model was 10%. Still, the rapid increase in CA over the course of the selection experiment across all lineages suggests that translational efficiency is a contributing factor shaping genome composition over time. The results of our simulations further support this conjecture. The experimental observations, in particular those of the J engineered lines, most closely fit models incorporating significant selection for more abundant host tRNAs (Fig. 4). The simulations uncover not only the landscape of mutations which could be explored by the engineered sequence but also the selective factors by which phage-host codon usage compatibility evolves. Similar to the results observed here, other studies which saw rapid virus-host codon compatibility recovery also observed fitness recovery^35,36^.

The extent to which virus and host sequences are compatibile varies between genes (Supplementary Fig. S1), suggesting that it is well-tuned at a genomic level. Just as viral codon deoptimization can reduce viral fitness, so too can optimization of natively ‘non-optimal’ genes^56^. In fact, genome manipulation alone is known to have fitness effects^57,58^. Nevertheless, the reduced fitness observed for the engineered S, E, and J strains created here and other studies of codon deoptimization^29–40^ are unlikely to be solely due to reduced translational efficiency. Codon engineering may lead to protein and mRNA misfolding or effect genome packaging, genome-capsid interactions, and protein-protein interactions. Even for the model bacteriophage ΦX174, many of the aforementioned processes are not fully understood. For instance, while it is known that some of the amino acids within the 66 bp targeted region of F interact with J, G, and F protein subunits during capsid formation^59^, only one nonsynonymous mutation (Q254H in the F coding sequence) occurred within a recognized protein-protein interaction site. Nonsynonymous mutations outside of the engineered region were observed in the evolved S, E, and J lines. This observation is not unique to the engineering of ΦX174 as other studies have likewise detected mutations outside of codon-modified segments^34–36^. The 12 nonsynonymous mutations and 10 nonsynonymous mutations in the S and E lines and the J lines, respectively, have not previously been observed in ΦX174 genomes. As these nonsynonymous mutations primarily occurred in structural proteins, they may have arisen in response to conformational changes in the engineered regions due to the initial molecular engineering and subsequent evolutionary response.

Isolating the contributions of selection for translational efficiency from those of translational accuracy, mutational bias, and drift has been the subject of decades of intense research activity^13,20–22,60–64^. Exploration of different phage-host systems provides a greater understanding into the evolution of codon usage within viruses. Using the ΦX174 system, we investigated translational efficiency in a small virus that does not encode its own tRNAs, as some larger phages do^65^, and is thus entirely dependent upon its host for biosynthesis. We observed rapid evolutionary responses that involved large increases in codon adaptiveness and fitness. While prior studies evolving codon-engineered phages have not observed such a rapid recovery^34^, we hypothesize that the rate of response is influenced by the genome itself – its size, topology, and composition. For instance, the physical constraints of single stranded genomes^66^ may contribute to the difference observed between the slow response observed in codon-modified T7 lines (dsDNA genomes) and the ΦX174 lines. The long-term evolution of the three engineered ΦX174 lines presented here provides the first empirical evidence of rapid selection for genome compatibility in a phage.

Exploring selection for virus-host genome compatibility in phages has two immediate benefits: it provides a model for engineering in viruses infective of eukaryotic cells (vaccine development) and engineering of phages for therapeutic use (phage therapy). The consistent increase in codons frequently utilized in the highly expressed genes of its host *E. coli* C suggests that translational efficiency was important during selection. The response observed was in all engineered strains and amongst all replicate lines. Thus, the consistent increase in virus-host genomic compatibility observed is a genome phenomenon rather than a residual of engineering within a specific region/gene. This is further supported through the computational simulations performed. The results provide insight into the tempo and mode in which viruses adapt in response to available hosts.

## Materials and Methods

### Calculation of codon usage

The complete sequence and annotation for the *E. coli* C genome (GenBank: NC_010468) was downloaded from NCBI. The codon frequencies were calculated for the 40 highly expressed gene sequences (HEGs)^19^. The unscaled proportion of codons in each codon family was calculated. In contrast to the relative synonymous codon usage^67^, this value, which we refer to as NRSCU^68^, weights each amino acid equally. NRSCU values were retrieved from the Codon Bias Database^68^.

The genome and annotation files for the viral species ΦX174 (GenBank: NC_001422), G4 (GenBank: NC_001420), α3 (GenBank: NC_001330), and ΦMH2K (GenBank: NC_002643) were downloaded from NCBI. Comparisons for the ΦMH2K-host codon compatibility also required the files, again retrieved from NCBI, for its host *Bdellovibrio bacteriovorus*; the reference genome for the strain HD100 was used (GenBank: NC_005363). Similarly, the codon usage of the HEGs within the *B. bacteriovorus* genome was calculated. Fig. S1 illustrates the phage-host codon usage compatibility (NRSCU value) for the six homologous coding regions of ΦX174, G5, α3, and ΦMH2K (panel A) and for all 11 homologous genes of ΦX174, G5, and α3 (panel B).

### Sequence design

Using the genome sequence for the ΦX174 Anc strain (GenBank: AF176034)^45^, the restriction enzyme cut sites within the F capsid coding region were identified with the NEB Cutter online tool^69^; PshAI and AhdI were selected because each recognized unique cut sites within the phage’s genome (at nucleotide positions 1694 and 1765, respectively). A 66 bp (nucleotide positions 1700–1765) region between these two cut sites, 22 codons, was then assessed for the individual codon usage within the *E. coli* C host species according to the codon bias of the HEGs from our calculations (Supplementary Table S1). Two sequences were designed, each containing eleven synonymous substitutions. The S strain includes only these eleven synonymous mutations while the E strain includes the synonymous mutations as well as a single nonsynonymous mutation at genome position 1718–1720. The nonsynonymous mutation of CGC (Arginine) for the least favored codon of Leucine was chosen as it is one of the least conserved residues within the region, as denoted by PDBsum’s residue conservation calculations^70,71^. The J strain targeted the region within the ΦX174 Anc strain, position 893–961. The restriction enzyme cut sites within the J coding region were also identified with the NEB Cutter online tool^69^; BstAPI and Sau961 were selected because each recognized cut sites flanking the coding region (at nucleotide positions 898 and 978, respectively). Just as had been performed for the design of the S and E strains, each codon in the J region targeted was compared to the NRSCU value for the *E. coli* genome (Supplementary Table S2). Only synonymous mutations were incorporated within the design of the engineered J strain. The oligos for the engineered sequences were synthesized by and obtained from Eurofins MWG Operon.

### Creation of engineered strain

The Anc strain was originally obtained from C. Burch (University of North Carolina, NC). This ancestral strain was plated from our freezer stock collection. One plate was harvested for the C line (control) and production of the engineered strains. Genomic extraction was performed using the UltraClean^TM^ Microbial DNA Isolation Kit following the standard protocol with an additional heating of the prep for 10 minutes at 70 °C to increase lysis efficiency (as suggested by protocol). Double digests using the corresponding enzymes for the F and J coding regions were conducted following the manufacturer’s protocol (New England Biolabs). The digested DNA was separated by gel electrophoresis through a 1.2% agarose gel. DNA fragments were excised from the gel and purified using the UltraClean^TM^ 15 DNA Purification kit. Ligation was performed with 7 μl of the digested DNA, 1 μl of the synthesized oligo, 1 μl ligase 10 × buffer, and 1 μl T4 DNA ligase overnight at 4 °C.

The ligation product (5 μl) was incubated with 400 μl of *E. coli* C spheroplast for 20 minutes at 37 °C; PAM medium (3 ml, pre-warmed to 37 °C) was added and the preparation was incubated for 90 min. The phage was released using a 1:10 dilution into water then titered. This process was carried out for each strain. Each phage strain’s lysate was then plated as follows: 100 μl of phage was added to 3 ml 0.5% agar LB and 1 ml of turbid *E. coli* C culture and then overlaid on a 1.7% agar LB plate. Plates were incubated overnight at 37 °C. Plates were harvested and suspended in 0.8% saline solution and treated with 50 μl chloroform. Single plaques were selected for each strain as the initial genotype for the subsequent lines. The genomes of the three engineered strains and the ancestral strain were confirmed by capillary sequencing.

### Propagation of engineered lines

The host *E. coli* C strain was also obtained from C. Burch (University of North Carolina, NC). Propagations were carried out as follows. One line of the S strain, four replicate lines of the E strain, three replicate lines of the J strain, and four lines of the ancestral strain (Anc) to serve as a control were propagated. While the S and E lines were propagated for 35 transfers, the J and Anc control lines were propagated for an additional 15 transfers. Co-cultures were carried out for seven hours per transfer. The emergence of bacterial resistance was also measured for co-culture of this duration determining that phage-sensitive *E. coli* dominated the population.

Initially LB was inoculated with the host *E. coli* C strain, taken from our frozen stock collection. 2 ml of turbid *E. coli* C cultures in exponential growth was aliquoted into a 13 mm culture tube along with 500 μl of phage solution titered such that the initial MOI < 0.001. (Under the conditions described hereafter, bacterial growth curves for our *E. coli* C strain were conducted – quantified both by spectrophotometry and colony counts – to ascertain phases of growth and CFU/mL throughout, results not shown.) The tube was then capped and placed in a shaking incubator at 37 °C for 7 hours after which the tube was treated with 200 μl of chloroform and gently vortexed for 5 seconds. Next, 500 μl was collected to inoculate freshly grown *E. coli* C in a new culture tube and 500 μl was collected into a microcentrifuge tube and stored at 4 °C. Every third transfer an additional 100 μl was collected and plated. Phage isolates were plated as described previously; virus lysates were stored both at −80 °C in 50/50 glycerol/water (v/v) as well as at 4 °C.

In an effort to maintain a static *E. coli* C population and thus minimize bacterial resistant to the phage from one transfer to the next, fresh *E. coli* C cultures were made daily from naïve cultures. Prior to inoculation with phage lysate, the naïve *E. coli* C culture was grown to the same density as the initial inoculations.

### Sequencing

Genomic extraction was performed of a single genotype per collection time using the UltraClean^TM^ Microbial DNA Isolation kit as described previously. Twelve primer pairs were designed using the Primer3 web-application^72^; when all twelve pairs are used, a minimum 2 × coverage of the genome is possible (primer sequences available upon request). PCR products were purified using ExoSAP-It and sequenced by the University of Chicago Cancer Research Center DNA Sequencing Facility.

Sequencing of the complete genome with a 4 × coverage was conducted after the 1^st^, 5^th^, 11^th^, 21^st^, and 35^th^ transfers for the S and E lines and after the 50^th^ transfer for the J lines. The C1 line was also sequenced after the1st 5^th^, 11^th^, 21^st^ and 35^th^ transfers. Sequences of the final evolved lines for the C1 line (GenBank: HM775306), S line (GenBank: HM775307), E1 line (GenBank: HM775308), E2 line (GenBank: HM775309), E3 line (GenBank: HM775310), and E4 line (GenBank: HM775311). The three J lines have been deposited as well (GenBank numbers being processed).

Sequencing at each collection time was conducted initially by extracting viral DNA from lysate. As such, the potential for numerous genotypes to be pooled existed. Additionally, we also plated via serial dilutions lysate collected and selected plaques at random for sequencing. In all cases the same genotype was recovered suggesting relatively low heterogeneity within the population.

### Sequence analysis

The sequences generated in this study were assembled using LaserGene SeqMan (DNASTAR, Inc.). Comparisons between the isolate contigs to the ancestral strain’s sequence were conducted by performing multiple sequence alignments using ClustalW within BioEdit (http://www.mbio.ncsu.edu/BioEdit/bioedit.html). Comparisons with environmental samples (Table S3; GenBank: AY751298, DQ079870-2, DQ079874-9907, DQ079909, NC_007817, NC_007821, and NC_007856)^73^ were also downloaded from NCBI.

### Codon adaptiveness

We used the unscaled proportion of codons in each codon family (NRSCU). This metric captures only increases/decreases in the use of host-preferred codons. These values are available through the CBDB site^68^. For each sequenced isolate, the codon adaptiveness or CA value was quantified. This metric represents the individual engineered line’s codon usage in comparison to this same window in the Anc strain relative to the codon usage within the host’s HEGs. This metric was implemented rather than the codon adaptation index or CAI value^74^ which takes length of the sequences into consideration; as we were comparing a region of the same length, length was not a contributing factor.

### Adsorption assays and burst assays

Plaque forming unit (PFU) counts were conducted by first titering the viral lysate (via dilution series conducted in triplicate) such that equivalent initial viral concentrations were plated: 100 μl of phage was added to 3 ml 0.5% agar LB and 1 ml of turbid *E. coli* C culture and then overlaid on a 1.7% agar LB plate. Each strain was plated with three replicates and plaques were counted. Adsorption assays were also performed, in triplicate per strain/line. The assay estimates fitness based on the doublings of phage concentration per hour which is not scaled to generation time which may differ among the engineered lines. This allows for a comparison between the Anc strain and evolved strains based on their absolute growth rate with their native host *E. coli* C. The assay is an additional measure of fitness and determines which phage can grow the fastest. *E. coli* C was grown for 90 minutes until visible turbidity was observed. 10 mL of *E. coli* C was inoculated with 1 mL of bacteriophage (titered such that MOI < 0.01) and incubated at 37 °C. After 5 minutes, 1 mL of the culture was removed, microcentrifuged, and the phage within the supernatant was plated via a dilution series; this represents the initial concentration of phage (*N*_o_). After 60 minutes (*t*), another 1 mL of the culture was removed and the phage in the supernatant was again isolated and titered. This is considered the final concentration of phage (*N*_*t*_). To find the adsorption rate (*k*), the equation ${N}_{t}={N}_{0}{e}^{-kCt}$ can be used where *C* is the bacterial cell density^75^. The experiments for determining the adsorption time can also be used to determine the burst size, taking just one pre-lysis data point and one post-lysis data point with multiple replicates^76^.

### Simulations

Each engineered line was evaluated separately. At each of the five (six in the case of the J lines) time points in which sequencing was performed, the same number of experimentally observed mutations – synonymous and nonsynonymous – were introduced. The CA was then calculated for the synthetically “evolved” sequence. Two strategies for mutation were developed. In the case of the first, nucleotides were mutated with equal probability of substitution for each of the four bases. The second strategy only incorporated a mutation if the change in the codon is for a tRNA that is more abundant in the *E. coli* host (as assessed via the NRSCU values of the two codons). Given the fact that such a small region of the genome was being investigated, the particular nucleotides targeted were selected at random (using Marsaglia’s CMWC strategy). Simulations were executed with 1000 replicates per time point per line accounting for varying influences (from 0–100%) of each of the two strategies. Simulations were performed using code developed here in C++ (available upon request).

### Data availability

Sequence data generated during the current study are available in the GenBank, accession numbers HM775306-HM775311. The three J lines have been deposited as well (GenBank numbers being processed). Sequences analyzed in this study are listed in Supplementary Table S3.

## Electronic supplementary material

Supplemental Figures

Table S1

Table S2

Table S3

Table S4

Table S5
