# Supplementary material for: The Evolution of Molecular Compatibility between Bacteriophage ΦX174 and its Host
Source: Sci Rep. 2018 May 29;8:8350. doi: 10.1038/s41598-018-25914-7 (PMC5974221; doi:10.1038/s41598-018-25914-7)
Supplement: Supplementary file 1 — Supplemental Figures [file 41598_2018_25914_MOESM1_ESM.pdf]

The Evolution of Molecular Compatibility between Bacteriophage ΦX174 and its Host

Alexander Kula, Joseph Saelens, Jennifer Cox, Alyxandria M. Schubert, Michael Travisano, Catherine Putonti

SUPPLEMENTARY FIGURES

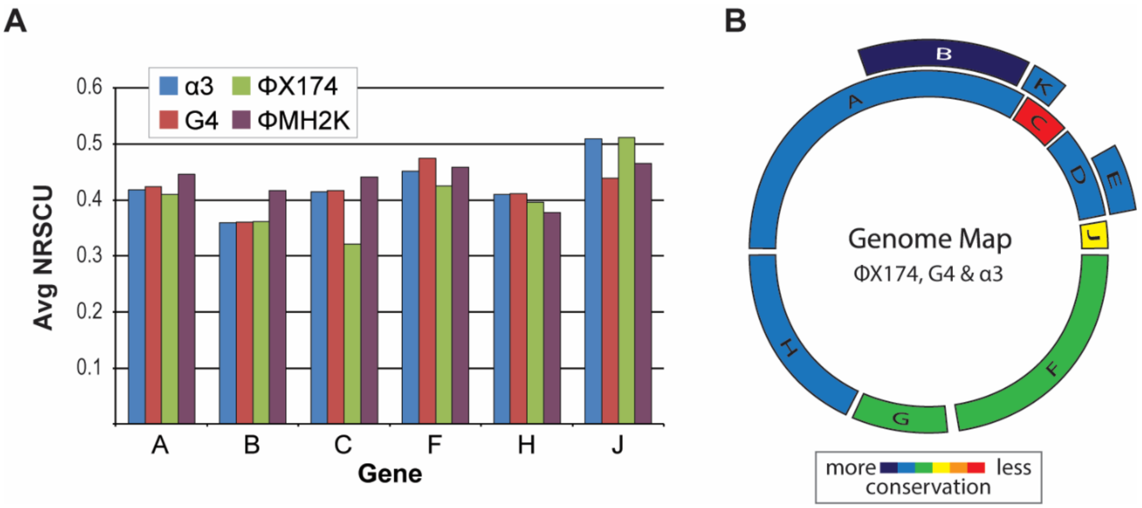

Fig. S1. Phage-host codon usage compatibility (NRSCU value) for the six homologous coding regions of the *Microviridae* RefSeqs ΦX174, G5, α3, and ΦMH2K **(A)** and for all 11 homologous genes of ΦX174, G5, and α3 **(B)**.

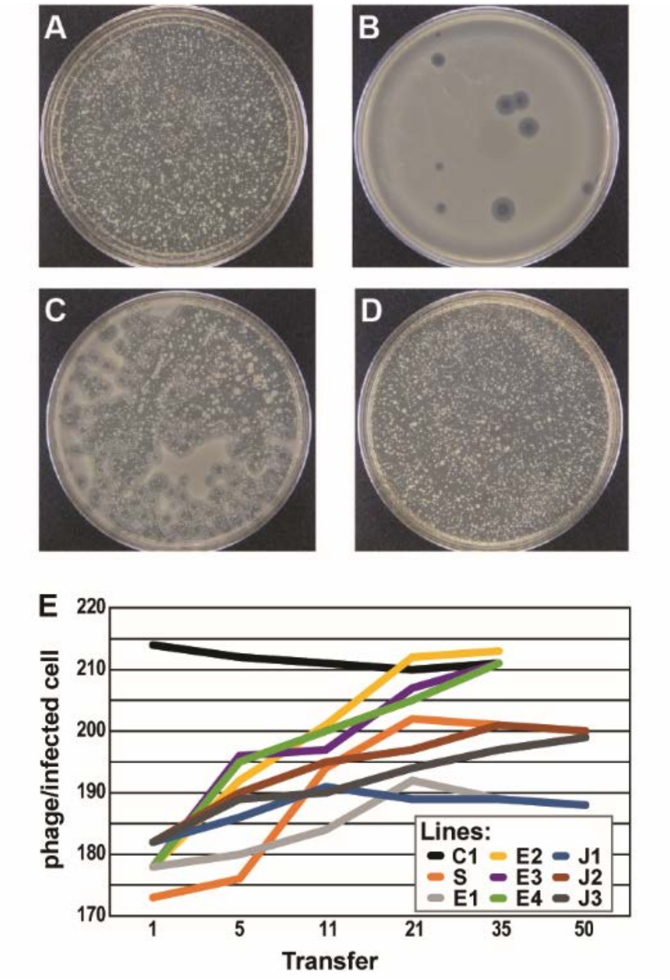

Fig. S2. Fitness response over the course of the selection experiment. PFU for C1 **(A)**, E1 after transfer 1 **(B)**, E2 after transfer 21 **(C)** and E2 after transfer 35 **(D)**. Panel **(E)** shows changes in burst size for C1, S, E1, E2, E3, and E4 lines.

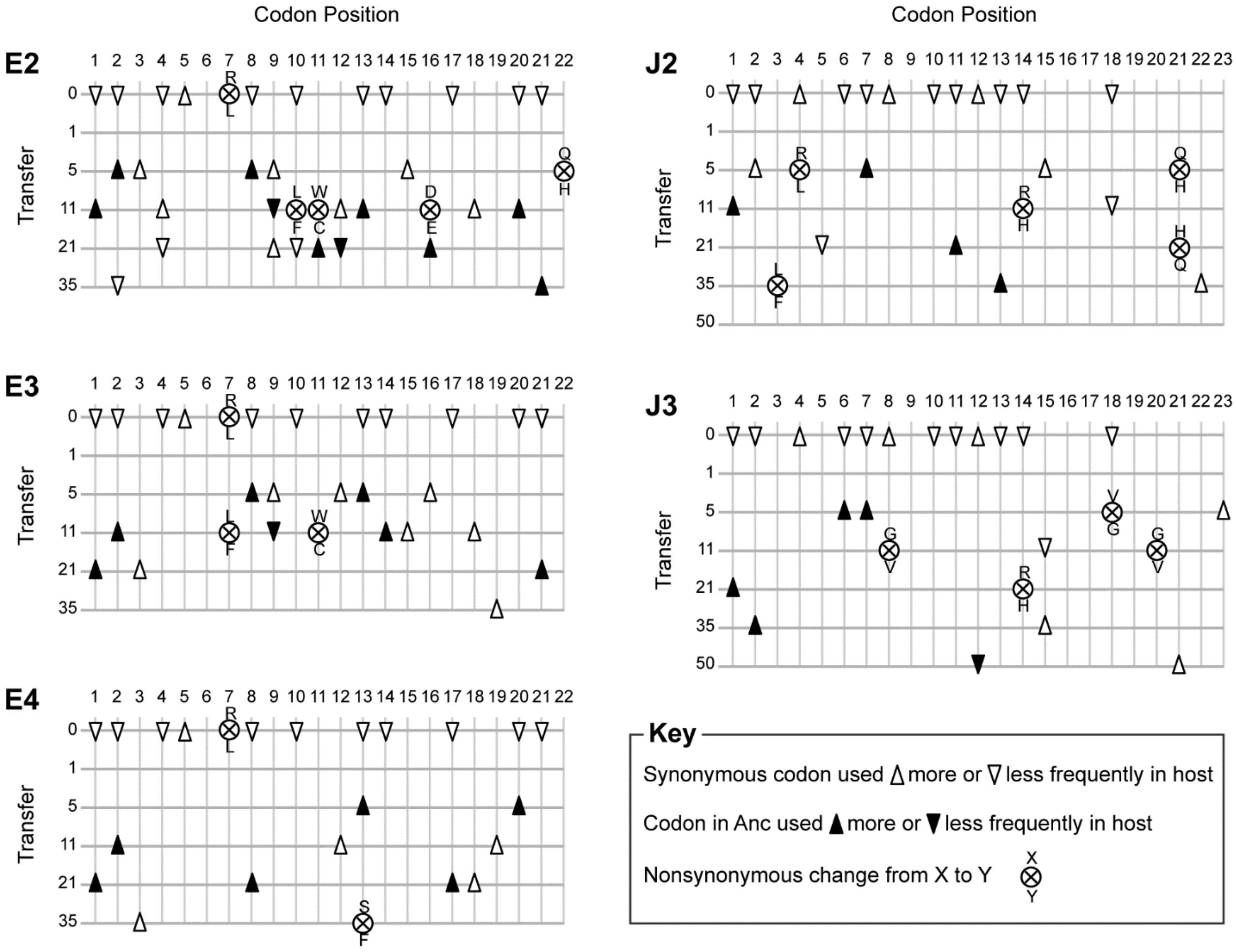

Fig. S3. Codon changes observed within the engineered lines (relative to the codon in the Anc strain) over the course of the selection experiment for E2, E3, E4, J2, and J3.

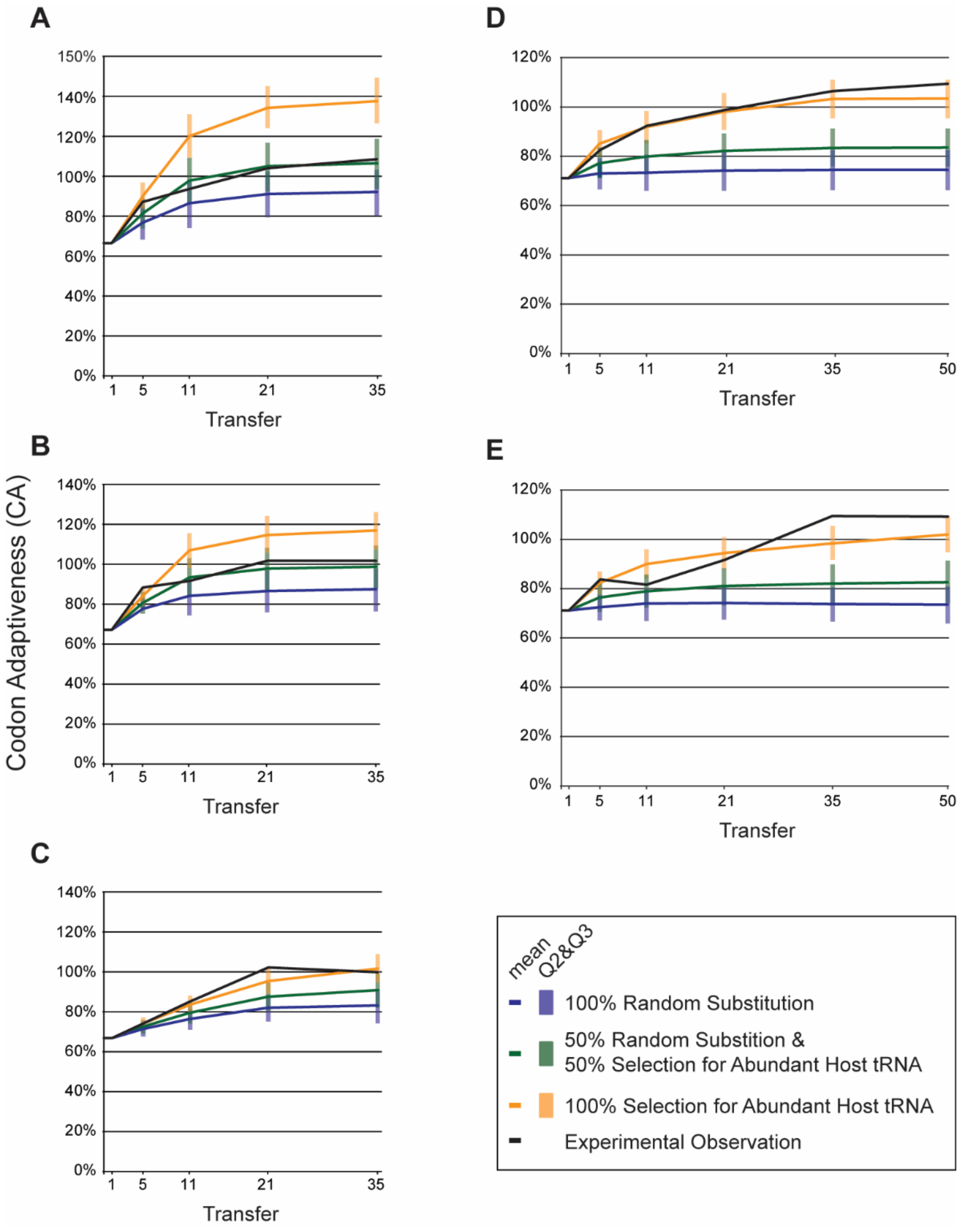

Fig. S4. Average CA values predicted over time from simulations under three different variations of the role of translational selection and random mutation and the CA values from the experimental assays for lines **(A)** E2, **(B)** E3, **(C)** E4, **(D)** J2, and **(E)** J3.
